# The burden of antenatal heart disease in South Africa: a systematic review

**DOI:** 10.1186/1471-2261-12-23

**Published:** 2012-03-30

**Authors:** David A Watkins, Motshedisi Sebitloane, Mark E Engel, Bongani M Mayosi

**Affiliations:** 1 Department of Medicine, Groote Schuur Hospital and University of Cape Town, J Floor Old Main Building, Groote Schuur Hospital, Observatory, Cape Town, 7925, South Africa; 2 Department of Obstetrics and Gynaecology, Nelson R Mandela School of Medicine, Congela, Durban, South Africa

## Abstract

**Background:**

Maternal mortality in South Africa is rising, and heart conditions currently account for 41 per cent of indirect causes of deaths. Little is known about the burden of heart disease in pregnant South Africans.

**Methods:**

We systematically reviewed the contemporary epidemiology and peripartum outcomes of heart disease in South African women attending antenatal care. Searches were performed in PubMed, ISI Web of Science, the EBSCO Africa-Wide database, the South African Union Catalogue, and the Current and Completed Research database (South Africa). References of included articles were also hand-searched. Studies reporting epidemiologic data on antenatal heart disease in South Africa were included. Data on morbidity and mortality were also collected.

**Results:**

Seven studies were included in the systematic review. The prevalence of heart disease ranged from 123 to 943 per 100,000 deliveries, with a median prevalence of 616 per 100,000. Rheumatic valvular lesions were the commonest abnormalities, although cardiomyopathies were disproportionately high in comparison with other developing countries. Peripartum case-fatality rates were as high as 9.5 per cent in areas with limited access to care. The most frequent complications were pulmonary oedema, thromboembolism, and major bleeding with warfarin use. Perinatal mortality ranged from 8.9 to 23.8 per cent, whilst mitral lesions were associated with low birth weight. Meta-analysis could not be performed due to clinical and statistical heterogeneity of the included studies.

**Conclusion:**

Approximately 0.6 per cent of pregnant South Africans have pre-existing cardiac abnormalities, with rheumatic lesions being the commonest. Maternal and perinatal morbidity and mortality continue to be very high. We conclude this review by summarising limitations of the current literature and recommending standard reporting criteria for future reports.

## Background

Globally, complications of heart disease during pregnancy account for a substantial proportion of maternal morbidity and mortality [1]. Physiological increases in blood volume, heart rate, and cardiac output occur in pregnancy but can exacerbate underlying cardiac conditions, particularly during the latter half of the pregnancy [2]. The prevalence of pre-existing heart disease amongst pregnant women worldwide varies but has been reported to range from 0.9 to 3.7 per cent [3]. Whereas congenital abnormalities are the commonest abnormalities found in pregnant women from industrialised regions, rheumatic heart disease still predominates in poorer countries [4].

South Africa is in the midst of an epidemiologic transition with a high burden of both cardiac diseases of poverty such as rheumatic heart disease (RHD) and chronic, non-communicable diseases such as hypertension [5]. Whilst the profile of cardiovascular disease is changing, maternal mortality in South Africa has quadrupled over the past decade, in part due to an ineffective health system [6]. The Ministry of Health's most recent survey on maternal deaths (2005-2007) found that, amongst "indirect" causes of death, heart diseases account for 41 per cent of cases. Seventy-seven percent of maternal deaths from cardiac disease occurred in those attending antenatal care, highlighting the need for improved management protocols for women with pre-existing heart disease [7].

Studies on the prevalence and outcome of heart diseases in pregnant South Africans (referred to as "antenatal heart disease" in this review) have reported varied findings depending on the times and settings in which they were performed. The primary objective of this review is to estimate the prevalence of cardiac disease during pregnancy and profile the various lesions that commonly afflict patients. The secondary objective of this review is to summarise maternal and foetal outcomes in this population.

## Methods

### Inclusion and exclusion criteria

Any study investigating antenatal heart diseases in pregnant South African women was considered for review. In order to obtain the best epidemiological data, only studies that were designed to measure hospital- or community-based burden of heart disease were included in the final analysis. Studies were excluded if they did not provide any information regarding a reference population of pregnant women without heart disease. The review was limited to studies conducted in participants over 18 years of age. Although we secondarily collected data on maternal and foetal outcomes, studies were not included or excluded on this basis. Language of publication was restricted to English articles only. Editorials and review articles were excluded.

### Search strategy

Two independent reviewers (DAW and MS) reviewed lists of articles obtained from several databases relevant to the South African population. The pre-specified search strategy for each database was as follows: MEDLINE was searched with the term "Heart Diseases"[MeSH] AND pregnan*[TIAB] AND "South Africa"[All Fields]; ISI Web of Science was searched with the term TS = HEART DISEASE (and) TS = PREGNAN* (and) CU = SOUTH AFRICA; and the EBSCO Africa-Wide database was searched with the term SU = heart or cardiac or cardiovascular (and) TI = disease* (and) SU = pregnan* (and) TX = South Africa. To search for South African conference proceedings, theses, and abstracts, two internal databases at the University of Cape Town Health Sciences Library were searched. Current and Completed Research (South Africa) [8] was searched using "heart AND pregnancy" and the South African Union Catalogue (SACat) section on South African Theses [8] was searched using the term ("heart" OR "cardiac") AND "pregnancy." All databases were accessed during the month of March 2011. Articles were selected on the basis of relevant title with relevant abstract and full text articles were obtained from potentially eligible reports. In the final stage of the search, reference lists of full-text articles were hand-searched. Discrepancies were resolved by consensus discussion between the two reviewers with adjudication by the senior author (BMM) as necessary.

### Data extraction

Two reviewers (DAW and MS) used a standardised data extraction form to obtain information on study design, patient demographics, and total numbers of each cardiac lesion. Secondarily, basic information was obtained on the rates of specific outcomes: maternal death, pulmonary oedema, thrombosis, haemorrhage, and foetal demise. Again, discrepancies were adjudicated by consensus discussion between the two reviewers with the assistance of the senior author (BMM) as necessary. When study data were incomplete or contradictory with regard to the primary objective, the original author of the manuscript was contacted to clarify his or her findings.

### Statistical terminology and analysis

Prevalences, case-fatality rates, and when applicable, perinatal mortality rates, are expressed per standard 100,000 deliveries. In hospital-based series and cohorts, we have reported the "prevalence" of antenatal heart disease as the number of patients in the study (all with heart disease) divided by the total number of deliveries at the institution, multiplied by 100,000. In some reports, cases of heart disease were reported in terms of "incidence"; however as most of the cardiac lesions likely predated pregnancy, we felt this term to be imprecise. We have also reported death rates as case-fatality in spite of the fact that papers tended to use the term "maternal mortality" because in our assessment, deaths were almost exclusively due to complications of the cardiac lesion (e.g., pulmonary oedema or coagulopathies related to warfarin misuse). We report "perinatal mortality" as any stillbirth, peripartum or neonatal death (i.e., less than 1 month). Unfortunately some studies did not have full records for all patients [9,10] and thus, we analysed perinatal deaths in a subset of women with cardiac disease, likely inflating mortality rates. We intended to perform a meta-analysis in an attempt to provide an aggregate of the prevalence data from the included studies. All reported numerical data were stored and analysed using Microsoft Excel 2010.

## Results

### Search results and study characteristics

**Figure 1:**
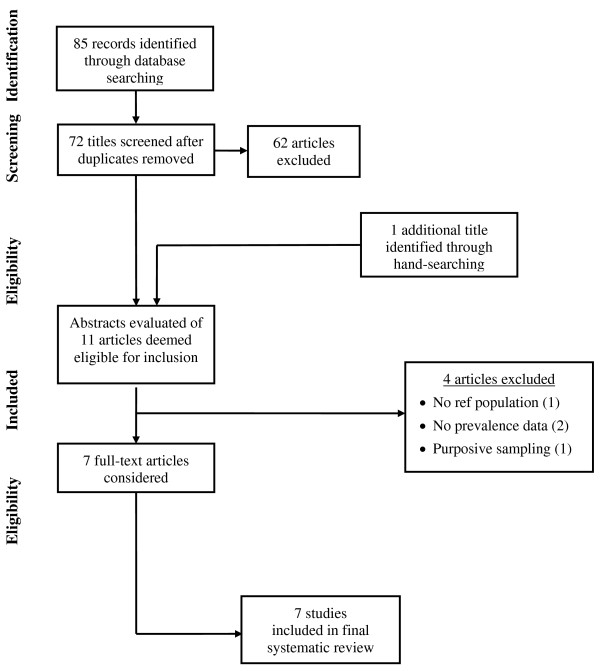
**Flow diagram of the article selection process**.

The search strategy and numerical results of the article selection process are shown in the flow diagram in Figure 1. MEDLINE retrieved 29 titles, ISI Web of Science 35 titles, EBSCO 18 titles, Current and Completed Research (South Africa) zero titles, and SACat (South Africa Catalogue) 3 titles. After screening the titles identified through the search and the removal of duplicates, 10 articles together with 1 additional article identified through hand-searching, were deemed eligible for inclusion. Four articles were excluded due to the absence of a reference population (n = 1), absence of prevalence data (2) and inadequate sampling (2); thus seven articles were included in the systematic review.

Summarised in Table 1 are the key design characteristics of the included studies. The earliest published study was conducted by Rush et al. (1979) [11], who reviewed a large database of hospitals comprising the Western Cape Peninsula maternity services, covering a 5 year period. All included patients had been evaluated in the tertiary cardiology clinic in consultation with an obstetrician. The next report of antenatal heart disease was published 20 years later, when Schoon et al. [12] collected all cases of cardiac disease in pregnancy who had delivered at Pelonomi Hospital (Bloemfontein) over 5 years. In 1999, Divanovic and Buchmann [13] performed an audit of all deliveries referred over 2 months to the tertiary obstetric unit of Chris Hani Baragwanath Hospital in Johannesburg. Their primary objective was to assess whether routine heart and lung auscultation in the clinic would increase the rate of diagnosis of cardiac or pulmonary disease; however, they were able to estimate the prevalence of heart disease as well.

**Table 1:** Characteristics of studies included in this review

Study ID (Year)	Location	Duration	Study design	*n*	Diagnosis type
Rush et al. [11]	Western Cape Province	5 yrs	R, C	697	NS
Schoon et al. [12]	Bloemfontein	5 yrs	R, T	164	X/E
Divanovic and Buchmann [13]	Johannesburg	2 mos	R, T	3191	X
Schoon [14]	Free State Province	2 yrs	P, C	42	X/E
Matlala [6]	Pretoria	3 yrs	R, T	95	E
Soma-Pillay et al. [10]	Pretoria	3 yrs	R, T	189	X/E
Nqayana et al. [15]	Durban	1 yrs	R, T	95	E

*yrs *years, *mos *months, *R *retrospective, *C *community-based, *T *based at tertiary hospital, *P *prospective, *X *physical examination diagnosis, *E *echocardiographic diagnosis, *NS *diagnostic method not specified. Diagnosis type refers to the techniques used to establish a final diagnosis in each patient. For example, in studies labeled X/E, either physical examination or echocardiography was permitted to establish a diagnosis

Schoon later wrote a PhD thesis [14] based on his initial observations on maternal heart disease in Bloemfontein. He undertook a prospective study over 2 years of all maternal deaths and severe morbidity due to heart disease, and then retrospectively screened all clinics and hospitals in the region for records of patients with cardiac disease who may have had uneventful deliveries during that period. The main objective of his study was to compare "complicated" cases of heart disease with these uneventful deliveries. Another thesis paper that met the inclusion criteria was published by Matlala [9], who performed a chart review of all patients with heart disease who delivered over a 3-year period at the Dr George Mukhari Hospital in Ga-Rankuwa, Pretoria. Similarly to the Schoon thesis, Matlala focused on maternal and perinatal outcomes but reported epidemiologic data as well.

Complementing the Matlala study, Soma-Pillay et al. [10] conducted a review of a maternal registry at Pretoria Academic Hospital, another major referral center in the Gauteng province of South Africa. This study was conducted over 4 years. Finally, Nqayana et al. [15] performed an audit of all cases of maternal heart disease over 1 year at Inkosi Albert Luthuli Central Hospital in Durban, which included a tertiary obstetric unit. Their cohort also included patients referred from King Edward VIII Hospital in Durban.

The data collected were almost exclusively retrospective and subject to a high degree of ascertainment bias. Our summary of the prevalence and outcomes data from the studies suggested that their highly variable results (see Table 2) were probably due to large heterogeneity in study design and size of the included studies (Table 1). Thus, although it was our original intention, we did not perform a meta-analysis.

**Table 2:** Prevalence of antenatal heart diseases; maternal and perinatal outcomes

Study ID (Year)	Prevalence	Case-fatality rate	Perinatal mortality rate
Rush et al. [11]	834	717	8895
Schoon et al. [12]	587	9146	-
Divanovic and Buchmann [13]	689	0	-
Schoon [14]	123	9524	23810
Matlala [6]	428	1980	9474
Soma-Pillay et al. [10]	943	3271	11640
Nqayana et al. [15]	616	0	9474

All figures are expressed per 100,000 deliveries and have been re-calculated based on manuscripts' raw data

### Epidemiology of heart disease in pregnancy

Table 2 contains data on the prevalence of antenatal heart disease as measured in each study. Values ranged from 123 to 943 per 100,000 deliveries, with a median prevalence of 616 per 100,000 deliveries. Of note, we excluded the "referred" sample of the Schoon thesis [14] as these data were not gathered in a community-based fashion and thus would have biased the results of the "index" sample. Also, we had to contact the senior author of the Nqayana et al. study [15] directly to obtain estimates of the number of deliveries at the included hospitals during that period, as these data were not reported in the original manuscript. Of note, during the review phase, we had a similar problem with a study on mitral stenosis in pregnancy [16]; this manuscript was eventually excluded due to lack of reference population data and concerns over purposive sampling.

The included studies tended not to focus on clinical history; however Schoon observed that the most common presenting symptoms of heart disease during the antenatal period were dyspnoea and paroxysmal nocturnal dyspnoea [14]. Nqayana et al. remarked that 28 per cent of their patients were previously unaware of their cardiac condition [15], whereas Divanovic and Buchmann reported new diagnosis in only 8 per cent [13].

Table 3 presents the commonest types of cardiac lesions found and their relative contribution to overall disease burden. Valvular heart disease, principally rheumatic mitral stenosis, mitral regurgitation, and surgically-repaired mitral valves, were the commonest type of lesions in all studies, ranging from 71 to 84 per cent. The exception was Schoon's thesis work [14], in which valvular lesions comprised 33 per cent and cardiomyopathies 55 per cent. Other studies reported cardiomyopathies as a minor condition on the same order as congenital disease, which ranged from 1 to 19 per cent but typically less than 10 per cent. The prevalence of "other" conditions ranged from 2 to 10 per cent and included conditions such as arrhythmia, pericarditis, and coronary disease, depending on the study.

**Table 3:** Proportion of cases with major types of heart disease as diagnosed in each study

Study ID (Year)	Native valve disease	Prosthetic valve	All valvular disease	Cardiomyo- pathies	Congenital disease	Other
Rush et al. [11]	70	1	71	1	19	9
Schoon et al. [12]	58	19	77	18	1	4
Divanovic and Buchmann [13]	41	32	73	8	14	5
Schoon [14]	29	5	33	55	2	10
Matlala [6]	54	29	83	4	5	7
Soma-Pillay et al. [10]	63	20	84	6	9	2
Nqayana et al. [15]	60	21	81	3	9	6

All values are expressed as percentages

### Maternal and foetal outcomes

Our secondary objective was to conduct a qualitative assessment of maternal and foetal outcomes. In the included reports, outcomes varied greatly, were generally centre-dependent, and improved over time. Case-fatality rates and perinatal mortality rates are reported in Table 2. Case-fatality rates ranged from 0 to 9,524 per 100,000 deliveries. One study reported that mortality was significantly higher in women with cardiac disease than in the pregnant population overall (717 vs 46 per 100,000) [11]. Of note, this was the only study that was conducted prior to the human immunodeficiency virus (HIV) era. Mitral stenosis, prosthetic valves, and cardiomyopathies were most frequently associated with maternal death [9-12], while isolated mitral regurgitation typically had a benign course [15]. Throughout the studies, the most frequent causes of death were pulmonary oedema, thrombosis or embolism, and haemorrhage, typically in the setting of anticoagulant use.

Case-fatality rates were highest in the studies originating from the Free State [12,14] and lowest in the Nqayana study [15] from Inkosi Albert Luthuli Central Hospital in Durban. Nqayana et al. attributed their superior outcomes to several factors, including the availability of percutaneous interventions, an intensive monitoring unit for pregnant women with cardiac disease, and a multidisciplinary team of obstetricians and cardiologists [15]. In the Free State, patients with isolated mitral stenosis tended to have favorable outcomes, in contrast to patients with cardiomyopathies. The study authors attributed this finding to "aggressive" prophylactic use of beta-blocking agents [12,14].

Maternal morbidity tended to follow globally established patterns and clear relations to different cardiac lesions. Decompensated heart failure was very common in cardiomyopathies (2 of every 3 patients) [12] and stenotic mitral lesions [10]. In the latter study, Soma-Pillay et al. reported pulmonary oedema in 84 per cent of their "near-miss" cases and reinforced the need for routine intensive care unit monitoring of cardiac patients in the peripartum period. Not surprisingly, even though thrombosis and hemorrhage were not uniformly fatal, they were common in all the studies and were thought to be related in part to variable adherence to anticoagulation as well as the intrinsic risks of warfarin use. Schoon et al., in particular, noted a 17-fold increase in maternal haemorrhage in patients on oral anticoagulation for prosthetic valves [12]. Similarly, Nqayana et al. reported 50 percent of patients with metalic valves had problems related to anticoagulation [15].

We also collected data on perinatal mortality, which ranged from 8,895 to 23,810 per 100,000 deliveries as noted in Table 2. Again several trends were noted. Firstly, antenatal heart disease is associated with a higher rate of foetal loss than in pregnant women without cardiac disease [9,11,15]. Furthermore, as Schoon noted, women with poor cardiac outcomes also had the highest perinatal mortality rates (19.3 per cent) when compared with uneventful pregnancies (no perinatal deaths noted) [14]. Secondly, mitral valve pathology tended to carry the highest risk for the foetus. Matlala, for example, noted a high incidence of low birth weight (< 2,500 g) deliveries in patients either with native stenosis or post mitral valve repair [9]; a finding that was corroborated by Nqayana et al [15]. Thirdly, indiscriminate prescription of warfarin was associated with a surprisingly high rate of foetal loss: Soma-Pillay et al. reported a 30 percent versus 5 percent foetal loss in mothers taking warfarin, presumably due to embryopathy. They attributed this situation to the unfortunate reality that most women taking warfarin are unaware of their pregnancy, or at least could not obtain a booking with a doctor until late in their first trimester after the effects of warfarin had peaked [10]. This late discovery of pregnancy was confirmed in Matlala's cohort [9].

### Psychosocial and public health aspects

Upon review of the studies, we found themes of poor access to care and adherence across the studies. For example, Schoon et al. noted a need for improved preconception counseling as well as access to sterilisation and contraception in mothers with a prior history of heart disease. They also remarked that improved detection of cardiac lesions in the primary health clinics is an "urgent priority" [12]. Similarly, Nqayana et al. stressed a need for earlier referral to specialist cardiac care for patients who are known to have cardiac conditions [15] Soma-Pillay et al. reviewed their "near-misses" and found that late booking, failure to book, and lack of self-awareness of the patient's condition were amongst the root causes for morbidity [10]. Unbooked patients in Matlala's study also tended to present late in the third trimester and with complications [9].

### Self-assessment of study quality

Finally, study authors typically noted potential biases in their research design, albeit inconsistently. Divanovic and Buchmann remarked that their recruitment method could have missed asymptomatic heart disease in the community that had not been referred to the tertiary hospital. They also speculated that undiagnosed heart disease may be more prevalent in rural areas that were not included in their study nor would have been as likely to be referred [13]. Conversely, Matlala duly noted the referral bias intrinsic in his maternal mortality calculations, as his study was conducted at a tertiary hospital [9]. Soma-Pillay et al. similarly noted their referral bias and also postulated (similarly to Divanovic and Buchmann) that some cases could have been missed [10]. Finally, in reporting the "population-based" prevalence of antenatal heart disease, Schoon noted the assumption that most women with heart disease seek care in the public health sector (the data to which he had access). He conceded that his recruitment method (based on case reporting) could miss a substantial proportion of cardiac disease that was asymptomatic or morbid but otherwise unreported [14].

## Discussion

We present the first systematic review of the burden of heart diseases in the obstetric population in South Africa. The unique nature of antenatal heart disease in South Africa presents special challenges for public health systems and clinical researchers alike.

### The changing profile of antenatal heart disease

Our review identified a variety of studies conducted over the past 30 years. Whilst the prevalence of antenatal heart disease appears to have been stable since 1979, the studies reviewed vary in their methods of diagnosis and in the outcomes observed. The literature appears to cover 3 distinct eras: the pre-HIV era, the 1990s prior to intensive monitoring units and high usage of balloon mitral valvuloplasty (BMV), and the present day. The role of echocardiography has evolved over the decades; in the earliest studies, echocardiography was not routinely available for diagnosis, which was usually made clinically or pathologically. In the modern era, echocardiography has become the standard of care and has allowed for very detailed documentation of cardiac lesions [15]. Regarding the role of the HIV/AIDS pandemic, no study has specifically investigated the interactions between HIV infection and antenatal heart disease; the exception being an analysis by Nqayana et al. demonstrating no difference in maternal or perinatal outcome based on HIV positivity, or even advanced disease (CD4 count < 200 cells/ml) [15].

Maternal mortality in South Africa has been rising over the past decade. Deaths due to HIV/AIDS, which is thought to be a major contributing factor to overall maternal mortality, increased 38 per cent from 2004 to 2007 [7,17]. Deaths from cardiac disease, however, increased 31 per cent, suggesting that the antenatal health system may be failing to detect and address the problem. Recent reports on maternal mortality have identified pervasive health systems failures such as lack of accountability, poor quality of care, lack of medical supplies and equipment, and endemic diseases themselves [6].

### Comparison to other studies internationally

The studies reviewed bear many similarities to contemporary reports from other developing regions, as well as differences to reports from industrialised nations. Studies in other countries report a global prevalence of antenatal heart disease on the order of 1 per cent, though there is some small variation by region [3,18]. Similar to other developing countries such as India [3], Egypt [19], and Turkey [20], as well as Nigeria [21] and Senegal [1],. the vast majority of cardiac lesions are rheumatic in origin, consistently ranging from 80 to 95 per cent of cases in these studies. There is a contrast to industrialised regions such as North America [18] and Europe [22], where the vast majority of lesions are congenital, in keeping with the almost total eradication of rheumatic heart disease from the Western world. However there is a uniquely high burden of cardiomyopathy in sub-Saharan Africa that is less common in other developing regions [23], a finding that is reflected in the studies reviewed (Table 3). Interestingly, studies from the Free State province reported a peculiarly high rate of cardiomyopathy even as compared with other provinces. The cause of this high rate is unclear and may reflect either a true epidemiological pattern or differences in terminology or diagnostic criteria.

Also in contrast to developed [24] and other developing [3,19,20] regions, but in keeping with other parts of sub-Saharan Africa [1], case fatality rates from antenatal cardiac disease (particularly mitral lesions) were quite high in South Africa, with a few exceptions. Rates were especially high for studies conducted in the Free State, which had a high proportion of rural cases as well as less access to the percutaneous interventions that have had great success in the referral hospitals in KwaZulu-Natal. Supporting the important role of access to mitral valve treatments, a prospective study of balloon mitral valvuloplasty (BMV) at King Edward VIII Hospital in Durban demonstrated no deaths out of 128 cases of mitral stenosis in pregnancy [16], in contrast to Schoon et al. in which 2 of 37 (5.4 per cent) of patients with mitral stenosis died [12]. Also of note, anticoagulation-related complications tended to be more common in South African studies as compared to studies from other developing regions, which report a high rate of appropriate anticoagulation management [19] and low prevalence of warfarin embryopathy [3]. This finding has important implications for local management guidelines regarding the timing of surgical repairs, recommendations for sterilisation, and choice of anticoagulants during pregnancy, which remains a controversial issue [9].

### Limitations of the studies reviewed

The present review was greatly limited by heterogeneity of the included studies, substantial biases in study design, and inconsistencies in reporting. As noted above, the most consistent data on cardiac diseases in pregnant South African women come from tertiary institutions, which carry an intrinsic referral bias particularly with regard to the identification of severe cardiac disease and a high risk of poor maternal and fetal outcomes. Thus we report estimates of "prevalence" while conceding that this term applies more appropriately to population-based research. Similarly, we have shown that the profile of antenatal heart disease has evolved over the past 3 decades, so we cannot be confident that these studies represent estimates of the same "true" prevalence.

With regard to reporting inconsistencies, we note that it was difficult for us to summarise details of cardiac lesions and outcomes as their descriptions were highly variable. For instance, some studies provided very fine detail of individual mitral valve lesions [15] while others simply reported the number of patients with "rheumatic heart disease." [10] Similarly, some studies [10,14] grouped outcomes according to the "near-miss" system [7], while others described outcomes in a meticulous case report format [11,15]. At times, we were unclear as to how diagnoses were made and the role of echocardiography [12]. One limitation that prohibited meta-analysis of our results was the unavailability of an exact baseline number of deliveries at one study site [15], and although the resulting estimate gave a prevalence consistent with other studies, this could not be considered a rigorous epidemiological study. In our opinion, these inconsistencies did not reflect individual study quality as much as the divergent objectives of the included studies. Thus, for future studies of antenatal heart disease that are conducted in South Africa, we recommend a minimum set of standardised reporting criteria as listed in Table 4.

**Table 4:** Recommended minimum standards for future observational studies on antenatal heart disease in South Africa

*Study parameters*	*Examples*
*Basic study information to include*	
- Study design/recruitment methods	
- Methods of diagnosis	Clinical examination, echocardiogram, or algorithm
- Basic demographics	Distribution by age and parity; proportion of pre-existing vs new diagnoses
- Data on the reference population	Total number of deliveries at the institution during the study period
*Profiling of cardiac lesions*	
- Detail valvular lesions	MS, MR, mixed mitral, AS, AI, any prosthetic (metal vs biologic) repairs or BMV
- Detail congenital lesions	Cyanotic, ASD, VSD (note any Eisenmenger syndrome)
- Detail cardiomyopathies	Peri- or post-partum vs idiopathic dilated vs other cardiomyopathies
- Mention other lesions	Arrhythmias (other than secondary AF), pericardial disease, coronary disease
*Profiling of outcomes*	
- Stratify mortality by lesion	Maternal death (include cause), foetal death (include cause if known)
- Stratify morbidity by lesion	Pulmonary oedema, thrombosis, haemorrhage, infective endocarditis, IUGR, SGA

*MS *mitral stenosis, *MR *mitral regurgitation, *AS *aortic stenosis, *AI *aortic insufficiency, *BMV *balloon mitral valvuloplasty, *ASD *atrial septal defect, *VSD *ventricular septal defect, *AF *atrial fibrillation, *IUGR *intrauterine growth restriction, *SGA *small for gestational age

At the systematic review level, we recognise that we are not only limited by the data in the studies themselves, but also by incomplete retrieval. We attempted to minimise this limitation by searching a wide variety of databases, including one Africa-wide database and two databases that focus on South African "grey" literature. Finally, there is a risk of reporting bias as some scientific observations might not have been published in indexed peer-reviewed journals.

### Recommendations for future work

This review represents an important step in advancing the science and clinical care of pregnant South Africans with heart disease. We have recommended certain standards for future research in this area, as outlined in Table 4. It is our hope that future studies on antenatal heart disease in South Africa and elsewhere will adhere to these recommendations and that these would ultimately provide data suitable for meta-analysis. We also offer our recommendations for other developing regions that have a similar profile of antenatal heart disease.

However, as noted in the latest Confidential Enquiry into maternal deaths in South Africa [7], better management guidelines are also needed regarding heart disease in pregnancy. Future guidelines will ideally be informed by a better evidence base than currently exists, taking into account the changing epidemiology of various lesions (and heart disease on the whole), new diagnostics and therapeutics, and psychosocial factors.

This study has a number of implications for policy, practice and research. First, it is clear that the majority of cases of antenatal heart disease in South Africa are sequelae of group A streptococcal infection, i.e., RHD. Control of rheumatic fever and RHD should be a top priority for the South African Ministry of Health, comprising primary and secondary prevention efforts [25]. A critical part of a comprehensive programme on RHD would also include awareness-raising amongst pregnant women: of their 27 new cardiac diagnoses, Nqayana et al. found that 18 were of RHD [15]. Similarly, Desai et al. found 42 per cent of their new diagnoses of (rheumatic) mitral stenosis were made during pregnancy [16]. Over time, prevention and education efforts could vastly reduce the number of adult women with valve damage and thus the absolute number of potentially morbid or fatal pregnancies. An additional point of advocacy in the field of child health relates to the consistent finding that, of all lesions, (rheumatic) mitral lesions typically confer the highest risk to the foetus [9,15,16].

Second, it is possible that screening for antenatal heart disease might improve case detection at the primary healthcare level and thus facilitate referral to specialist care. Echocardiography-based screening of pregnant women has been proposed as a potential modality, though its cost-effectiveness is unclear [26,27]. B-type natriuretic peptide has also been shown to be an effective biomarker for cardiac disease in pregnant women and correlates with adverse outcomes [28]. In light of the poor sensitivity of auscultation and data that suggest it is ineffective for screening in routine antenatal care [13], prospective studies are needed to determine the utility of alternative screening modalities and their cost-effectiveness. Ideally, a multicentre, prospective observational study of antenatal heart disease in South Africa could be conducted at a population level to simultaneously gather epidemiological data (using echocardiography as a "gold standard") and assess several potential screening modalities.

Third, more clinical trials are needed to determine the appropriate use of certain cardiac treatments during pregnancy. The evidence for BMV as a palliative measure appears strong and percutaneous valve repair and replacement seems to be on the horizon [29]. More rigorous trials of medical strategies specific to the South African environment are needed, particularly to manage complications of mitral stenosis such as atrial fibrillation and heart failure. Perhaps most importantly, we have shown consistently poor maternal and foetal outcomes for patients taking warfarin during pregnancy. We believe that better anticoagulation guidelines for pregnant women are needed and, as part of this, newer and potentially less dangerous alternatives to warfarin [30] must be explored.

Finally, there remain substantial psychosocial barriers and health disparities that need to be investigated and rectified. Rheumatic heart disease is a disease of poverty [31] and most of the pregnant women included in these studies were of lower socioeconomic status. Women in such conditions are less aware of their health and also, less empowered to take care of themselves in terms of early booking and adherence to medications, as noted above [10].

## Conclusions

Heart disease affects at least 0.6 per cent of pregnant women attending antenatal care in South Africa and contributes to the persistently high maternal and perinatal mortality seen in this country. Rheumatic heart disease remains the predominant condition and often results in adverse outcomes despite optimal management. We have highlighted key limitations of the current literature and have recommended minimum reporting criteria for future studies in this area. Despite the inherent management complexities of antenatal heart disease, a renewed focus on these issues will advance maternal and child health in South Africa.

## Abbreviations

RHD: Rheumatic heart disease; HIV/AIDS: Human immunodeficiency virus/acquired immune deficiency syndrome; BMV: Balloon mitral valvuloplasty

## Competing interests

The authors declare that they have no competing interests.

## Authors' contributions

DAW conducted the search, analysed the results, and drafted the manuscript. MS conducted the search and analysed the results. MEE developed the search strategy and reviewed the data for suitability of meta-analysis. BMM conceived of the study, participated in its design and coordination, and helped to draft the final manuscript. All authors read and approved the final manuscript.

## Pre-publication history

The pre-publication history for this paper can be accessed here:

http://www.biomedcentral.com/1471-2261/12/23/prepub

## References

[B1] Diao M Kane A Ndiaye MB Mbaye A Bodian M Dia MM Sarr M Monsuez JJ Ba SA Pregnancy in women with heart disease in sub-Saharan Africa Arch Cardiovasc Dis 2011 104 6-7 370 374 10.1016/j.acvd.2011.04.001 21798468

[B2] Reimold SC Rutherford JD Clinical practice. Valvular heart disease in pregnancy N Engl J Med 2003 349 1 52 59 10.1056/NEJMcp021265 12840093

[B3] Bhatla N Lal S Behera G Kriplani A Mittal S Agarwal N Talwar KK Cardiac disease in pregnancy Int J Gynaecol Obstet 2003 82 2 153 159 10.1016/S0020-7292(03)00159-0 12873775

[B4] Sawhney H Aggarwal N Suri V Vasishta K Sharma Y Grover A Maternal and perinatal outcome in rheumatic heart disease Int J Gynaecol Obstet 2003 80 1 9 14 10.1016/S0020-7292(02)00029-2 12527454

[B5] Sliwa K Wilkinson D Hansen C Ntyintyane L Tibazarwa K Becker A Stewart S Spectrum of heart disease and risk factors in a black urban population in South Africa (the Heart of Soweto Study): a cohort study Lancet 2008 371 9616 915 922 10.1016/S0140-6736(08)60417-1 18342686

[B6] Moszynski P South Africa's rising maternal mortality is due to health system failures, says report BMJ 2011 343 d5089 10.1136/bmj.d5089 21824903

[B7] Saving mothers 2005-2007: fourth report on confidential enquiries into maternal deaths in South Africa. Department of Health Pretoria, South Africa 2009

[B8] Databases available by subscription from the University of Cape Town Health Sciences Library http://www.lib.uct.ac.za/medical/Accessed 28 March 2011

[B9] Matlala MP Outcome of pregnancy in cardiac patients at Dr. George Mukhari Hospital M.Med. thesis for the Department of Obstetrics and Gynaecology, Faculty of Medicine, University of Limpopo; published 2005

[B10] Soma-Pillay P MacDonald AP Mathivha TM Bakker JL Mackintosh MO Cardiac disease in pregnancy: a 4-year audit at Pretoria Academic Hospital S Afr Med J 2008 98 7 553 556 18785398

[B11] Rush RW Verjans M Spracklen FH Incidence of heart disease in pregnancy. A study done at Peninsula Maternity Services hospitals S Afr Med J 1979 55 20 808 810 462330

[B12] Schoon MG Bam RH Wolmarans L Cardiac disease during pregnancy - a Free State perspective on maternal morbidity and mortality S Afr Med J 1997 87 1 C19 C22 9186451

[B13] Divanovic E Buchmann EJ Routine heart and lung auscultation in prenatal care Int J Gynaecol Obstet 1999 64 3 247 251 10.1016/S0020-7292(99)00006-5 10366046

[B14] Schoon MG Severe morbidity and mortality associated with cardiac disease during pregnancy in the Free State Public Health Service PhD thesis for the Faculty of Health Sciences, University of the Orange Free State; published 2001

[B15] Nqayana T Moodley J Naidoo DP Cardiac disease in pregnancy Cardiovasc J Afr 2008 19 3 145 151 18568175 PMC3974559

[B16] Desai DK Adanlawo M Naidoo DP Moodley J Kleinschmidt I Mitral stenosis in pregnancy: a four-year experience at King Edward VIII Hospital, Durban, South Africa BJOG 2000 107 8 953 958 10.1111/j.1471-0528.2000.tb10395.x 10955424

[B17] Saving Mothers 2002-2004: third report on confidential enquiries into maternal deaths in South Africa. Department of Health Pretoria, South Africa 2006

[B18] Siu SC Sermer M Colman JM Alvarez AN Mercier LA Morton BC Kells CM Bergin ML Kiess MC Marcotte F Prospective multicenter study of pregnancy outcomes in women with heart disease Circulation 2001 104 5 515 521 10.1161/hc3001.093437 11479246

[B19] Abdel-Hady ES El-Shamy M El-Rifai AA Goda H Abdel-Samad A Moussa S Maternal and perinatal outcome of pregnancies complicated by cardiac disease Int J Gynaecol Obstet 2005 90 1 21 25 10.1016/j.ijgo.2005.03.008 15913623

[B20] Madazli R Sal V Cift T Guralp O Goymen A Pregnancy outcomes in women with heart disease Arch Gynecol Obstet 2009 281 1 29 34 19326134 10.1007/s00404-009-1050-z

[B21] Abengowe CU Das CK Siddique AK Cardiac failure in pregnant Northern Nigerian women Int J Gynaecol Obstet 1980 17 5 467 470 6103844 10.1002/j.1879-3479.1980.tb00190.x

[B22] Stangl V Schad J Gossing G Borges A Baumann G Stangl K Maternal heart disease and pregnancy outcome: a single-centre experience Eur J Heart Fail 2008 10 9 855 860 10.1016/j.ejheart.2008.07.017 18760667

[B23] Sliwa K Damasceno A Mayosi BM Epidemiology and etiology of cardiomyopathy in Africa Circulation 2005 112 23 3577 3583 10.1161/CIRCULATIONAHA.105.542894 16330699

[B24] Silversides CK Colman JM Sermer M Siu SC Cardiac risk in pregnant women with rheumatic mitral stenosis Am J Cardiol 2003 91 11 1382 1385 10.1016/S0002-9149(03)00339-4 12767443

[B25] Mayosi B Robertson K Volmink J Adebo W Akinyore K Amoah A Bannerman C Biesman-Simons S Carapetis J Cilliers A The Drakensberg declaration on the control of rheumatic fever and rheumatic heart disease in Africa S Afr Med J 2006 96 3 Pt 2 246 16610104

[B26] Veasy LG Tani LY Minich L The logic for extending the use of echocardiography beyond childhood to detect subclinical rheumatic heart disease Cardiol Young 2009 19 1 30 33 10.1017/S1047951109003540 19154628

[B27] Zuhlke L Mayosi BM The challenge of screening for asymptomatic rheumatic heart disease in South Africa SA Heart 2009 6 2 100 103

[B28] Tanous D Siu SC Mason J Greutmann M Wald RM Parker JD Sermer M Colman JM Silversides CK B-type natriuretic peptide in pregnant women with heart disease J Am Coll Cardiol 2010 56 15 1247 1253 10.1016/j.jacc.2010.02.076 20883932

[B29] Smith CR Leon MB Mack MJ Miller DC Moses JW Svensson LG Tuzcu EM Webb JG Fontana GP Makkar RR Transcatheter versus surgical aortic-valve replacement in high-risk patients N Engl J Med 2011 364 23 2187 2198 10.1056/NEJMoa1103510 21639811

[B30] Mega JL A new era for anticoagulation in atrial fibrillation N Engl J Med 2011 365 11 1052 1054 10.1056/NEJMe1109748 21870977

[B31] Watkins DA Zuhlke LJ Engel ME Mayosi BM Rheumatic fever: neglected again Science 2009 324 5923 37 19342571 10.1126/science.324.5923.37b

